# Supplementary material for: Development of a Novel Stress and Immune Gene Panel for the Australasian Snapper (Chrysophrys auratus)
Source: Genes (Basel). 2024 Oct 29;15(11):1390. doi: 10.3390/genes15111390 (PMC11594050; doi:10.3390/genes15111390)
Supplement: Supplementary file 1 [file genes-15-01390-s001.zip › genes-3255787-supplementary.pdf]

Table S1. Search strategies and results

Database	Search strategy	Results	Search date
Web of Science all*	TOPIC: ("Pagrus auratus" OR "Chrysophrus auratus" OR "Pagrus major " OR "Sparus aurata" OR "Tamure Sparus") AND TOPIC: (immun*) and refined with review papers	1905 results refined with review papers (approx.110 papers)	01/04/2020
Web of Science all*	TOPIC: ("Pagrus auratus" OR "Chrysophrus auratus") AND TOPIC: (immun*) AND TOPIC: (gene* OR "T cell" OR pentraxin OR cytokines OR TNF OR IFN OR CXC OR iNOS OR COX)	34 results	01/04/2020
Web of Science all*	TOPIC: ("Pagrus major " OR "Sparus aurata" OR "Tamure Sparus") AND TOPIC: (immun* or cytokines) AND TITLE: (gene*)	198 results	01/04/2020
Google Scholar	(gene* OR "T cell" OR pentraxin OR cytokines OR TNF OR IFN OR CXC OR iNOS OR COX) and ("Pagrus major " OR "Sparus aurata" OR "Tamure Sparus" OR bream OR "chrysophrys auratus) AND "gene expression" AND immunology	6 results from 50 hits (Limited to first 5 pages over previous 2 years)	06/04/2020

*Web of Science all includes Web of Science core, current content connect, CABI, FSTA, MEDLINE, Russian Science Citation Index, SciElO Citation Index
